# Radiographic Knee Osteoarthritis Identification Using Wearable Plantar Pressure: Secondary Cross-Sectional Analysis of Interpretable Framework Integrating Biomechanical Descriptors and the Multiscale Synergy Index

**DOI:** 10.2196/101026

**Published:** 2026-09-11

**Authors:** Guanyu Xin, Hongbo Yao, Jingyuan Qiao, Huilin Yao, Siyuan Li, Lu Liu, Jingyi Zhang, Ke Qin, Lin Shu

**Affiliations:** 1 School of Future Technology, South China University of Technology, 777 Xingye Avenue East, Panyu District, Guangzhou, Guangdong, 511442, China, 86 13128120219

**Keywords:** radiographic knee osteoarthritis, plantar pressure, gait biomechanics, Plantar Synergy Index, subject-level identification, feature-selection stability

## Abstract

**Background:**

Radiographic knee osteoarthritis (ROA) is associated with abnormal plantar loading and altered gait coordination. Insole-based plantar-pressure sensing offers a practical wearable approach to ROA identification, but existing methods typically use either conventional biomechanical descriptors or end-to-end temporal models. An interpretable subject-level framework that captures both local abnormalities and distributed coordination is, therefore, needed.

**Objective:**

This study aimed to develop an interpretable subject-level framework that combines conventional biomechanical descriptors with coordination-level plantar information to identify ROA.

**Methods:**

In secondary cross-sectional analysis, using a previously reported Pearl River Osteoarthritis Cohort plantar-pressure dataset from our group, we analyzed 92 participants (43 with ROA and 49 without ROA; mean age 63.0, SD 8.4 y). The source cohort was recruited from clinical and nearby community sources at Zhujiang Hospital of Southern Medical University, Guangzhou, China, between January 2022 and February 2023. We developed an interpretable participant-level classification framework integrating conventional biomechanical descriptors with a coordination-level Plantar Synergy Index (PSI). Participant-wise stratified split allocated 72 participants for 5-fold cross-validation, and 20 for locked independent evaluation. Feature selection used Fisher-score ranking (k=50). All point estimates are accompanied by 2000-iteration bootstrap 95% CIs, and paired model comparisons used DeLong tests. All statistical tests used α=.05. Additional analyses examined feature stability, phase-resolved PSI patterns, robustness to perturbation and design variation, and comparisons with representative end-to-end temporal baselines.

**Results:**

The fusion model achieved the best primary classification performance on the independent test set, with an *F*_1_-score of 0.842 (95% CI 0.615‐1.000) and accuracy of 0.850 (95% CI 0.700‐1.000). It also showed balanced performance across additional metrics, with area under the receiver operating characteristic curve (AUROC) of 0.818 (95% CI 0.576‐1.000), precision of 0.800 (95% CI 0.538‐1.000), recall of 0.889 (95% CI 0.636‐1.000), and specificity of 0.818 (95% CI 0.571‐1.000). DeLong testing showed that fusion outperformed PSI-only at the AUROC level (*P*=.02). Stable retained descriptors were dominated by biomechanical features, with a reproducible PSI component. Phase-resolved analyses suggested distributed stance-phase coordination differences. Robustness and baseline comparisons supported the stability of the framework, but the wide CIs indicate that the findings remain preliminary.

**Conclusions:**

This study introduces an interpretable participant-level representation that integrates local biomechanical loading with multiscale interregional temporal coordination, extending plantar-pressure approaches based on summary descriptors or end-to-end models. Across evaluated settings, the framework showed competitive identification and preliminary robustness. Local biomechanical descriptors constituted the main discriminative basis, whereas PSI contributed a reproducible coordination-level component whose incremental predictive value remains to be established. This broadens plantar-pressure analysis from local loading to distributed coordination. External multicenter prospective validation is required before evaluating the framework as an imaging adjunct for functional assessment and longitudinal monitoring.

## Introduction

Osteoarthritis is a chronic disease characterized by whole-joint degeneration. It causes pain, reduced activity, and functional decline, and is a major global cause of disability [1]. Knee osteoarthritis is one of the most common forms, and its burden continues to rise, highlighting the need for scalable and repeatable assessment tools that can capture functional change and support rehabilitation management and longitudinal follow-up [2].

In clinical practice, imaging—especially X-ray—is widely used to assess knee osteoarthritis, and the Kellgren-Lawrence (K-L) scale is one of the most common radiographic grading systems [3]. Here, we focus on radiographic knee osteoarthritis (ROA), using radiographic status as the reference standard for classification-oriented model development and evaluation. However, radiographic grades mainly reflect static structural changes and do not directly capture functional abnormalities during walking and daily activities. Moreover, a mismatch between imaging findings and symptoms, including pain, has been reported repeatedly [4,5]. Together, these limitations suggest that imaging alone may be insufficient for rehabilitation-oriented functional assessment, motivating the exploration of wearable functional biomarkers that reflect gait-related impairment [1].

Gait biomechanics is closely linked to the functional manifestations of knee osteoarthritis [6]. Recent studies have further linked ROA-related functional impairment to altered plantar-pressure behavior, gastrocnemius muscle-tendon-unit stiffness, and 3D gait biomechanics [7,8]. Plantar pressure is particularly informative because it reflects both load distribution and gait control, providing interpretable measures such as pressure distribution, peak pressure, and center-of-pressure progression. Wearable insole systems are especially attractive due to their portability and ease of deployment, enabling data collection in both clinical and daily-life settings [9-12]. Earlier intelligent-footwear research demonstrated daily-life monitoring of spatial and temporal plantar pressure, in-shoe conditions, center-of-pressure trajectories, and acceleration in people with diabetes [12]. Recent digital insole studies further suggest promising potential for detecting ROA-related gait signatures [13].

Current plantar-pressure modeling for knee osteoarthritis largely follows 2 paradigms, each with distinct strengths and limitations. The first relies on handcrafted biomechanical features, which are typically interpretable but often emphasize localized loading characteristics or outcome-level summaries. As a result, they may not adequately capture how plantar regions coordinate and covary over time during gait. The second applies end-to-end deep learning directly to pressure sequences, which is more flexible for temporal pattern learning but can be less stable and less interpretable in medical settings with limited sample sizes [14]. However, within plantar-pressure–based ROA identification, an important unresolved challenge is how to represent pressure signals in a way that preserves biomechanical interpretability while also capturing distributed interregional coordination beyond conventional summary descriptors.

Previous plantar-pressure studies have identified ROA-related abnormalities in anatomically meaningful foot regions, including reduced hallux loading, altered heel pressure distribution, and increased mediolateral center-of-pressure excursion [15-18]. However, most studies have relied on summary-level biomechanical descriptors and have not examined how plantar regions coordinate with one another over the gait cycle. On the same 92-participant Pearl River Osteoarthritis Cohort (PROC) cohort, previous work by our group distinguished ROA from non-ROA using handcrafted features at the window level [19] and predicted functional performance at the participant level [20], but neither incorporated interregional coordination structure. Whether coordination-level information captured from the same plantar-pressure signals provides discriminative power beyond conventional biomechanical features therefore remains an open question—one that the growing adoption of wearable-based biomechanical assessment makes increasingly timely [21].

This evidence, across both the broader literature and our own previous work, points to a specific representation gap in plantar-pressure–based ROA classification, that is, existing frameworks either provide interpretable biomechanical anchors or flexible temporal modeling, but none explicitly incorporate interregional coordination structure. In this study, we treat conventional biomechanical descriptors and interregional coordination patterns as 2 complementary components of plantar-pressure representation. Accordingly, in this secondary methodological analysis of a previously reported plantar-pressure cohort, we aimed to determine whether multiscale plantar coordination information, quantified by the Plantar Synergy Index (PSI), provides participant-level discriminatory information beyond conventional biomechanical descriptors for ROA identification. We hypothesized that combining PSI with biomechanical descriptors would improve threshold-dependent classification performance while preserving interpretability under a strict participant-wise, leakage-controlled evaluation protocol. To test this hypothesis, we developed an interpretable framework and evaluated it using a locked independent test set, feature-stability analyses, phase-resolved PSI visualization, robustness analyses, and comparisons with representative end-to-end temporal baselines.

## Methods

### Study Design, Data Source, and Participants

#### Study Design and Data Source

This study was a secondary cross-sectional analysis of a plantar-pressure dataset previously reported in related studies [19,20]. The analytic dataset was derived from the PROC and included 92 participants, comprising 43 participants with ROA and 49 participants with non-ROA. ROA was defined according to the original radiographic criterion used in the source study, namely modified K-L grade ≥2.

The source cohort was recruited from the PROC study at Zhujiang Hospital of Southern Medical University, Guangzhou, China, between January 2022 and February 2023. Participants in the source PROC cohort were recruited from osteoarthritis outpatient clinics and nearby communities; therefore, the recruitment procedure is best characterized as nonprobability clinical or community-based recruitment rather than random population sampling. No new participant recruitment or additional plantar-pressure data collection was conducted for the secondary analysis of this study. This study used the available bilateral in-shoe plantar-pressure recordings and ROA status for participant-level ROA identification.

A participant-wise stratified split was used for model development and final evaluation, allocating 72 participants to the development set and 20 participants to the locked independent test set. The *Journal Article Reporting Standards* (JARS) participant flowchart documenting cohort flow, data completeness, and the development or test split is provided in Figure S1 in Multimedia Appendix 1.

#### Eligibility and Participant Characteristics

The original source cohort involved adults with knee osteoarthritis who were able to walk independently, as described in the related cohort report [20]. For the analysis of this study, inclusion required available bilateral wearable in-shoe plantar-pressure recordings, available ROA or non-ROA status, and complete demographic and anthropometric variables, including age, height, weight, and BMI.

All 92 participants in the available source dataset met these analytic inclusion requirements. Baseline demographic and anthropometric characteristics stratified by ROA status are reported in Table S1 in Multimedia Appendix 1. The ROA group was older than the non-ROA group (mean 66.6, SD 7.5 y vs mean 59.8, SD 7.9 y; Cohen *d*=−0.885; Mann-Whitney *P*<.001) and had higher BMI (mean 25.4, SD 3.6 kg/m^2^ vs mean 23.7, SD 3.1 kg/m^2^; Cohen *d*=−0.504; Welch *t* test *P*=.02). Height and weight did not differ significantly between groups. Individual-level sex distribution and K-L grade were not available in the dataset of this analysis and are therefore reported descriptively based on the available source information rather than used as adjustment covariates.

#### Measures and Covariates

The primary outcome was binary ROA status, defined as modified K-L grade ≥2 for the classification task of this study. The primary predictors were participant-level plantar-pressure descriptors derived from conventional biomechanical features, PSI-derived plantar coordination features, or their fusion. Detailed preprocessing, windowing, PSI construction, biomechanical feature definitions, and participant-level aggregation procedures are described in the subsections under the Methods section and Multimedia Appendices 2–4. Available demographic and anthropometric covariates included age, height, weight, and BMI.

#### Sample Size, Power, Precision, and Missing Data

The analytic sample size was determined by the available PROC source dataset rather than by a new prospective sample-size calculation. The final analytic sample included all 92 available participants. Given the modest sample size, especially the 20-participant locked independent test set, we emphasized estimation precision rather than relying on point estimates alone. CIs and paired area under the receiver operating characteristic curve (AUROC) comparisons were used to support precision-aware interpretation, as described in the Statistical Analysis subsection.

No data were missing; all 92 participants completed the full bilateral plantar-pressure acquisition, and no pressure windows were missing. The 4 demographic and anthropometric variables used in this analysis, namely age, height, weight, and BMI, were complete for all participants.

#### Overlap Statement With Previous Cohort Studies

This study uses the same 92-participant PROC plantar-pressure dataset previously used in related studies by our group [19,20]. It overlaps with those studies in participant cohort, wearable in-shoe pressure-sensing system, bilateral raw plantar-pressure recordings, and available clinical or radiographic source information. There is also partial authorship overlap with the previous cohort publications.

However, this study differs in analytical objective, feature representation, outcome modeling, and evaluation protocol. Previous work focused on conventional biomechanical descriptors for window-level ROA classification [19] or participant-level functional-performance prediction [20]. In contrast, this study evaluates whether multiscale PSI-derived plantar coordination descriptors provide complementary participant-level information when combined with conventional biomechanical descriptors under a strict participant-wise, leakage-controlled classification protocol. A structured comparison with previous plantar-pressure and gait-based studies is provided in Multimedia Appendix 5.

Accordingly, the contribution of this manuscript is methodological and representational. No new cohort, device, deployment setting, or longitudinal validation is claimed.

### Plantar-Pressure Acquisition and Preprocessing

Plantar-pressure data were collected using a bilateral in-shoe wearable system, with 1 insole per foot. Each insole contained 8 discrete pressure sensors, yielding 16 channels sampled at 20 Hz. The detailed 16-channel sensor-to-anatomy mapping and region definitions are provided in Multimedia Appendix 2.

**Figure 1.**
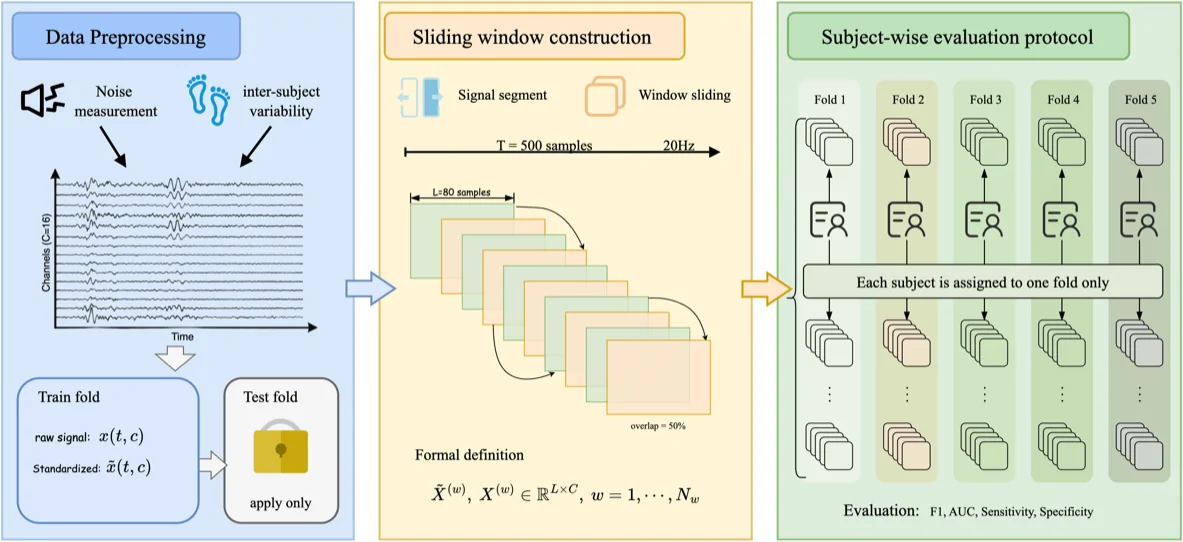
Leakage-controlled data preprocessing pipeline. All windows from the same participant were kept within a single subset throughout the workflow. Representation construction, preprocessing, and model development were performed without using information from the independent test set.

Figure 1 illustrates the leakage-controlled preprocessing and evaluation pipeline used in this study. The full workflow consisted of following 3 steps: participant-wise data splitting, construction of parallel signal representations, and sliding-window construction. All windows from the same participant were assigned to the same subset throughout, preventing repeated-participant leakage across model development and final testing and reducing optimistic bias in performance estimation [22,23]. We first performed a participant-level stratified split, yielding an independent test set of 20 participants (11 non-ROA and 9 ROA) and a development set of 72 participants. All subsequent model-development procedures were confined to the development set.

For each participant, a fixed-length steady-walking segment of 500 samples was retained from the original plantar-pressure recording. From this same segment, 2 signal representations were constructed for subsequent analyses—the raw plantar-pressure signal in its original scale, and a participant-wise channel-wise standardized representation. Let $\mu_{c}$ and $\sigma_{c}$ denote the mean and SD of channel c computed from that participant’s steady-walking signal. We first standardize each channel within a participant:


(1) $$\begin{array}{c} \tilde{x}\left(t,c\right)=\frac{x\left(t,c\right)-\mu_{c}}{\sigma_{c}+\epsilon } \end{array}$$

where $\epsilon =10^{-6}$ ensured numerical stability. The same representation-construction procedure was applied to both the development and independent test sets. After representation construction, the retained steady-walking segment was segmented into overlapping windows to capture short-term dynamics. We set the window length to L=80 samples (4 s at 20 Hz) and the stride to s=40 samples (2 s), yielding 50% overlap. This duration was chosen to span approximately 3‐4 complete gait cycles at typical walking cadences (1.0‐1.2 Hz), balancing within-window stability for PSI estimation against temporal resolution. This procedure produced $N_{w}=11$ windows per participant, so that all participants contributed the same number of window-level samples. Each window was denoted as $X^{\left(w\right)}\in R^{L\times C}$ for $w=1,\ldots ,N_{w}$.

### Window-Level Feature Extraction

**Figure 2.**
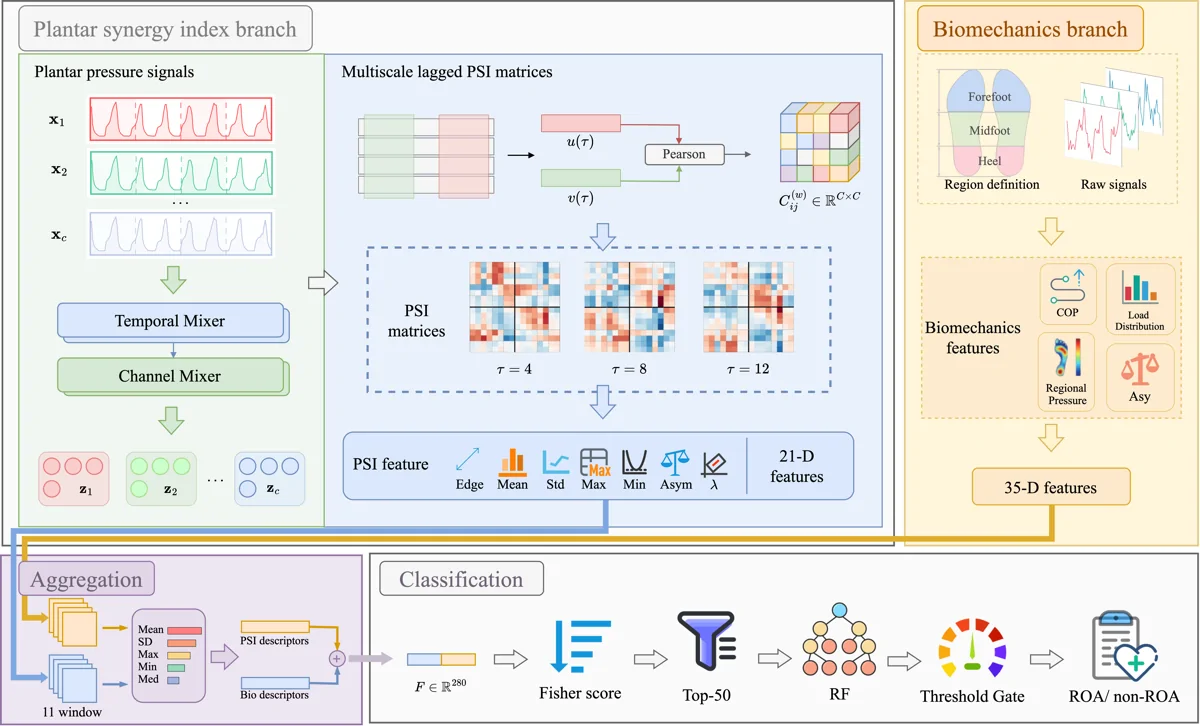
Two-branch plantar-pressure representation framework for subject-level ROA (radiographic knee osteoarthritis) identification. The biomechanics branch operates on raw pressure windows, whereas the PSI (Plantar Synergy Index) branch operates on the subject-wise standardized representation. Window-level features from both branches are aggregated to subject level for feature selection and final classification.

We extracted 2 complementary window-level feature sets from the windowed plantar-pressure signals (ie, conventional biomechanical features and PSI-based coordination features; Figure 2). In the PSI branch, we aimed to capture distributed coordination patterns across plantar regions that may not be adequately characterized by conventional biomechanical descriptors alone. The PSI-related analyses were based on the participant-wise standardized representation. Estimating PSI directly from the window ${\tilde{X}}^{\left(w\right)}$ can be sensitive to sensor noise, gait-phase misalignment, and interparticipant amplitude variation, particularly in small cohorts. To improve robustness while preserving a lightweight formulation, we introduced a mixer-style representation transform inspired by the token-channel mixing strategy of multilayer perceptron (MLP)-mixer and its time-series adaptations [24,25].

Within each development split, this transform was pretrained using only non-ROA windows from the corresponding training subset in a self-supervised one-step-ahead prediction task, consistent with predictive representation learning for sequential data [26,27]. The resulting module alternates temporal and channel mixing to produce a stable intermediate representation before PSI construction. Specifically, the transformed signal was defined as:


(2) $$Z^{\left(w\right)}=M_{2}\;\left(M_{1}\;\left({\tilde{X}}^{\left(w\right)}\right)\right),\;Z^{\left(w\right)}\in R^{L\times C}$$

where M1 and M2 implement temporal mixing and channel mixing, respectively, using lightweight MLPs shared across channels and time steps to control complexity. PSI matrices were then constructed for each window over a multiscale lag set T = {4, 8, 12}, chosen to span short-to-long coordination offsets under the current sampling rate. The sensitivity of this design choice was further examined in the design-variation analyses. Each PSI matrix was defined from lag-aligned interregional correlation, motivated by the lead-lag structure commonly observed in plantar-pressure signals [28,29]. For window w, let z_i^(w)(t)^ denote channel i at time t in Z^(w)^. For any τ ∈ T, we formed a lead-lag aligned signal pair:


(3) $$\begin{array}{c} \begin{array}{c} u_{i}^{\left(w\right)}\left(\tau \right)={\left[z_{i}^{\left(w\right)}\left(1+\tau \right),\ldots ,z_{i}^{\left(w\right)}\left(L\right)\right]}^{⊤},\\ v_{j}^{\left(w\right)}\left(\tau \right)={\left[z_{j}^{\left(w\right)}\left(1\right),\ldots ,z_{j}^{\left(w\right)}\left(L-\tau \right)\right]}^{⊤} \end{array} \end{array}$$

and defined the PSI matrix $C^{\left(w\right)}(\tau )\in R^{C\times C}$ as


(4) $$\begin{array}{c} C_{ij}^{\left(w\right)}\left(\tau \right)=corr\left(u_{i}^{\left(w\right)}\left(\tau \right),v_{j}^{\left(w\right)}\left(\tau \right)\right),\tau \in T \end{array}$$

where corr(⋅,⋅) is Pearson correlation over length L-τ.

From each PSI matrix $C^{\left(w\right)}(\tau )$, we extracted a compact set of PSI-derived summary features to characterize global coupling structure at each lag scale. These features capture complementary aspects of PSI structure, including edge density, distributional summaries (mean, SD, maximum, minimum), lagged asymmetry, and dominant spectral organization. The full definitions, mathematical formulations, and naming rules for PSI are provided in Multimedia Appendix 3. Thus, PSI features from all 3 lag scales were included in the candidate representation space before downstream feature selection.

Meanwhile, the biomechanics branch computed conventional plantar-pressure features directly from the raw pressure window $X^{\left(w\right)}$, providing interpretable anchors such as overall statistics, center-of-pressure (CoP), load ratios, and asymmetry indices. The complete feature list (35 features), channel mappings, region definitions, and formulas are provided in the Multimedia Appendix 2. This branch outputs window-level biomechanics feature vector $f_{\text{Bio}}^{\left(w\right)}\in R^{35}$. For each window, the PSI and biomechanics features were concatenated to form the window-level feature vector $f^{\left(w\right)}=\left[f_{\text{PSI}}^{\left(w\right)};f_{\text{Bio}}^{\left(w\right)}\right]$.

### Participant-Level Modeling

To obtain 1 risk score per participant, we aggregated window-level features into a participant-level representation using fixed summary statistics. For a scalar window feature sequence $\{u_{k}^{\left(w\right)}{\}}_{w=1}^{N_{w}}$, we computed 5 statistics (mean, SD, maximum, minimum, and median) and stacked them as


(5) $$\begin{array}{c} A_{k,m}=Stat_{m}\left({\left\{u_{k}^{\left(w\right)}\right\}}_{w=1}^{N_{w}}\right),m=1,\ldots ,5 \end{array}$$

Applying this aggregation to PSI features $f_{\text{PSI}}^{\left(w\right)}\in R^{21}$ and biomechanics features $f_{\text{Bio}}^{\left(w\right)}\in R^{35}$ yielded $F\in R^{105}$ and $G\in R^{175}$, respectively, and the final participant-level feature vector was $x=[F;G]\in R^{280}$.

Given the limited cohort size relative to the 280-dimensional representation, the resulting feature space was prone to redundancy and overfitting. We therefore used Fisher score ranking as a simple filter-based selection step to retain the most discriminative participant-level features. For feature i, the score was defined as


(6) $$\begin{array}{c} Fscore\left(i\right)=\frac{{\left(\mu_{i,1}-\mu_{i}\right)}^{2}+{\left(\mu_{i,0}-\mu_{i}\right)}^{2}}{{\left(\sigma_{i,1}\right)}^{2}+{\left(\sigma_{i,0}\right)}^{2}+\epsilon } \end{array}$$

Here, $\mu_{i,c}$ and $\sigma_{i,c}$ denote the class-specific mean and standard deviation for class c∈{0,1}, $\mu_{i}$ denotes the pooled mean, and $\epsilon =10^{-6}$ is a small constant for numerical stability. The Fisher score quantifies between-class separability relative to within-class variance. The top-50 setting was fixed before locked independent test-set evaluation according to the development protocol. The top-k sensitivity analysis reported below was conducted to examine whether this feature count lay within a stable performance range, rather than to select the final feature count using the locked test set.

### Leakage-Controlled Model Development and Evaluation

The downstream classifier was a random forest (RF), chosen for its robustness in small-sample settings and its ability to capture nonlinear feature interactions [30]. To avoid information leakage in threshold selection, the operating threshold was determined within the development set by participant-level 5-fold cross-validation. In each fold, all model-development steps, including Fisher score ranking, top-50 retention, and RF fitting, were performed using only the fold-specific training partition. Each validation participant contributed 1 aggregated participant-level feature vector and 1 corresponding RF probability as an out-of-fold estimate, ensuring that the probability used for threshold selection was generated without using that participant in representation learning, feature selection, or classifier fitting. The final threshold was selected from these pooled out-of-fold predictions to maximize the score.

After this internal development step, Fisher score ranking was reapplied once to the full development set to construct the final top-50 panel, and the RF classifier was refit on all 72 development participants using the locked feature panel and locked operating threshold before 1-time evaluation on the independent test set. Thus, cross-validation was used for internal model development rather than for reporting averaged final performance, whereas the independent test set was reserved for a single locked evaluation. Further implementation details are provided in Multimedia Appendix 4.

We additionally benchmarked the proposed fusion model against 3 representative end-to-end temporal baselines to examine how it compares with alternative modeling paradigms under the same fixed-length input, development or test split, and participant-level evaluation protocol. InceptionTime was included as a strong convolutional baseline for time-series classification [31], Medformer as a Transformer baseline whose multigranularity patching design is well-suited to medical time-series classification [32], and TodyNet as a graph-based baseline that directly models multivariate temporal dynamics through dynamic graph construction and temporal graph learning [33]. For fairness, each baseline was trained with its own tuned model-specific hyperparameter configuration, while the shared data handling and evaluation setup are summarized in Multimedia Appendix 4.

### Statistical Analysis

All evaluations were performed at the participant level, with 1 ROA risk score and 1 binary decision assigned to each participant. We used the *F*_1_-score as the primary metric:


(7) $$\begin{array}{c} F1=\frac{2\cdot Precision\cdot Recall}{Precision+Recall} \end{array}$$

In addition to F1, we reported AUROC, sensitivity, specificity, and precision to provide a more comprehensive characterization of discrimination performance. Given the limited test-set size (n=20), point estimates alone could overstate differences between models. We therefore computed 2000-iteration bootstrap 95% CIs for all metrics, and supplemented the bootstrap analysis with paired DeLong tests to evaluate whether observed AUROC differences were statistically distinguishable from chance. To assess whether the primary model performance could be explained primarily by available demographic and anthropometric variables, we conducted a supplementary model-level covariate sensitivity analysis. First, we trained a demographics-only RF model using age, height, weight, and BMI under the same participant-wise evaluation protocol. Second, we added these variables to the Fusion feature set before Fisher-score selection and RF classification. The complete covariate sensitivity analysis protocol is detailed in Multimedia Appendix 6.

To better understand which participant-level descriptors contributed consistently under resampling, we examined feature-selection stability across the 5 development folds. Within each fold, Fisher score ranking was applied to the training partition using the same feature-selection procedure as in the main pipeline. For each candidate feature, we summarized the number of folds in which it was retained and its mean within-fold Fisher score. For interpretation, retained features were further grouped by descriptor family, lag scale, and anatomical sensor region according to the insole topology.

To characterize robustness, we evaluated the fusion, bio-only, and PSI-only models under the following 3 perturbation categories: temporal scaling, random channel dropout, and additive Gaussian noise. Temporal scaling was tested at 0.8×, 1.0×, and 1.2× relative to the native sampling rate. Random channel dropout was evaluated at masking rates (0.0, 0.1, 0.2, and 0.3), and additive Gaussian noise was evaluated at σ=0.00, 0.01, 0.03, and 0.05 applied to the standardized signal. Design sensitivity was further examined by varying the sparsity threshold in PSI matrix construction, the lag configuration (single-scale vs multiscale), and the window length using 3 s, 4 s, and 5 s windows with 50% overlap. Phase-resolved PSI patterns were visualized by aligning trajectories to the normalized stance phase using 5% bins and pooling them at the group level within each functional module and lag setting. These perturbation and design-sensitivity analyses were used to assess the stability of the selected configuration and were not used to reselect the feature count, window length, lag configuration, sparsity threshold, classifier, or operating threshold.

### Ethical Considerations

The study was approved by the Institutional Review Board (IRB) of Zhujiang Hospital, Southern Medical University, Guangzhou (IRB 2019-KY-016‐02). All participants were enrolled from the PROC study and provided written informed consent before data collection; the original consent permitted secondary analysis of deidentified data without additional consent. Participants received financial compensation of approximately US $30 per participant. No identifiable images of individual participants appear in this manuscript or its supplementary materials.

## Results

### Primary Classification Performance

We first compared PSI-only, bio-only, and fusion models under the leakage-controlled development or test protocol. As summarized in Table 1, PSI-only showed limited standalone classification performance, with a low *F*_1_-score, AUROC, accuracy, and precision despite relatively high recall. Bio-only provided a strong baseline with substantially improved discrimination and a balanced sensitivity-specificity profile. Fusion showed numerically higher *F*_1_-scores (0.842 vs 0.778), accuracy (0.850 vs 0.800), precision (0.800 vs 0.778), and recall (0.889 vs 0.778) than bio-only, while AUROC and specificity were identical between the 2 models.

**Table 1.** Participant-level classification performance on the independent test set for PSI^a^-only, bio-only, and fusion models.

Metric	PSI-only	Bio-only	Fusion
*F*_1_-score	0.609	0.778	0.842
AUROC^b^	0.525	0.818	0.818
Accuracy	0.550	0.800	0.850
Precision	0.500	0.778	0.800
Recall	0.778	0.778	0.889
Specificity	0.364	0.818	0.818

a PSI: Plantar Synergy Index.

b AUROC: area under the receiver operating characteristic curve.

**Figure 3.**
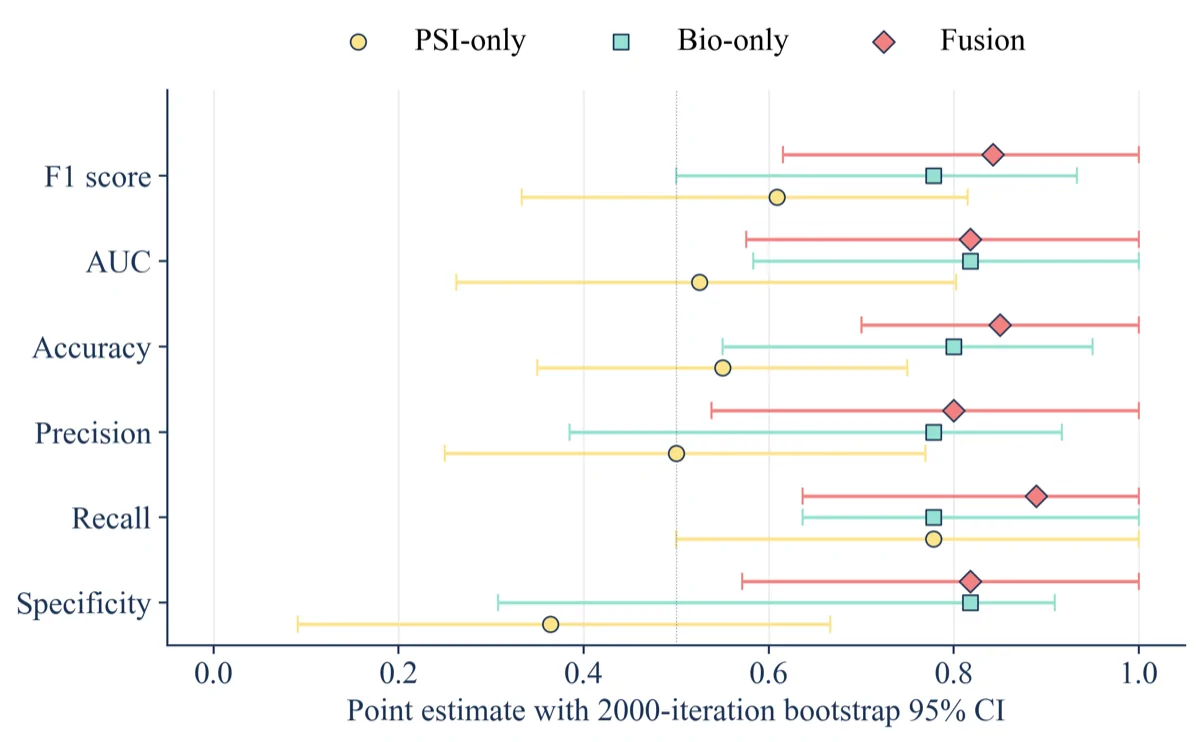
Bootstrap 95% CIs for six metrics across PSI (Plantar Synergy Index)-only, Bio-only, and Fusion (2000 iterations, n=20). Point estimates and CIs are shown for each branch–metric combination; wide and overlapping intervals reflect the limited test-set size. These intervals support cautious interpretation of between-branch differences, particularly for Fusion versus Bio-only. AUC: area under the curve.

Bootstrap 95% CIs for all metrics are shown in Figure 3. The intervals were wide and overlapping across branches; for instance, the *F*_1_-score spanned 0.500‐0.948 for bio-only and 0.615‐1.000 for fusion. Paired DeLong tests showed that both fusion and bio-only outperformed PSI-only at the AUROC level (*P*=.02 and *P*=.04, respectively), whereas fusion did not differ from bio-only in AUROC (*P*>.99). Together, these results suggest that PSI alone is insufficient as a standalone classifier, while fusion showed a directionally favorable threshold-dependent profile relative to bio-only. Given the wide and overlapping CIs, this pattern should be interpreted cautiously as preliminary evidence that PSI may complement biomechanical descriptors rather than as definitive superiority over bio-only. The complete bootstrap confidence intervals and paired DeLong comparisons are provided in Multimedia Appendix 7.

The covariate sensitivity analysis is summarized in Multimedia Appendix 6. The demographics-only RF model performed substantially worse than the fusion model, indicating that age, height, weight, and BMI alone could not reproduce the primary plantar-pressure–based classification performance. Adding these demographic and anthropometric variables to the fusion feature set did not materially improve the *F*_1_-score, AUROC, or specificity. Within the augmented feature space, age and BMI were retained by Fisher score ranking, whereas height and weight were not retained. These findings suggest that the fusion model was not simply a demographic proxy, although residual confounding cannot be excluded.

### Cross-Fold Feature-Selection Stability and Representative Stable Descriptors

**Figure 4.**
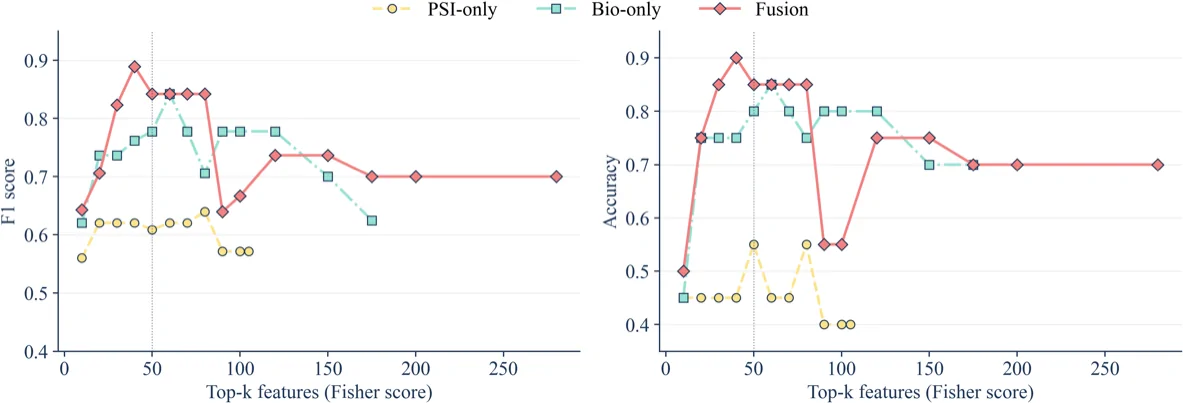
Top-k sensitivity scan for Fisher-score feature retention. Left: *F*_1_-score across retained feature counts (k ∈ {10, ., 280}). Right: Accuracy across retained feature counts. PSI (Plantar Synergy Index)-only, Bio-only, and Fusion are shown in each panel. The analysis evaluates whether the prespecified Top-50 configuration lies within a stable performance range, rather than serving as final model tuning.

To examine whether the locked top-50 feature-retention setting lay within a stable performance range, we conducted a top-k scan across 15 values; results are illustrated in Figure 4. Fusion performance at k=50 (*F*_1_-score=0.842) lay within a high-performance plateau, with similarly strong *F*_1_-score and accuracy at neighboring k values. At smaller k values, performance was limited by an insufficient number of retained features, whereas larger panels around k>=90‐100 showed degraded classification performance, with *F*_1_-score dropping to 0.667 at k=100. These results suggest that k=50 was located within a relatively stable operating range rather than representing an isolated favorable point. However, this analysis was used to assess the robustness of the selected configuration and was not used for final feature-count tuning.

To evaluate whether the descriptors selected during the 5-fold development procedure were reproducible under sample variation, we analyzed feature-selection stability across the 5 development folds. In each fold, Fisher score ranking was applied to the fold-specific training partition using the same selection procedure as in the main pipeline, and the top 50 descriptors were retained. Each descriptor was then summarized by its cross-fold selection rate and mean within-fold Fisher score, and descriptors retained in at least 3 of 5 folds were regarded as stable. This cross-fold view was used for interpretation because, in the present small-sample setting, the single top-50 panel obtained from the full development set may be sensitive to sample composition, whereas repeatedly retained descriptors are more likely to reflect reproducible discriminative structure [34-36].

**Figure 5.**
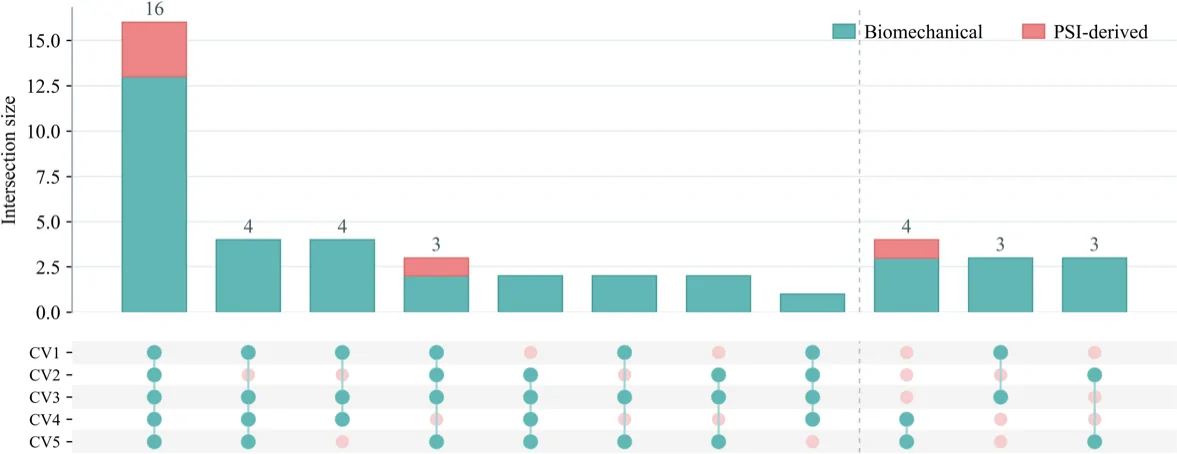
Cross-fold overlap structure of selected descriptors across the 5 development folds. Each column shows one descriptor-overlap pattern across the 5 development folds. Bar height indicates the overlap size, and stacked colors indicate the numbers of biomechanical and PSI (Plantar Synergy Index)-derived descriptors. For clarity, only a subset of lower-overlap patterns (<3 folds) is displayed. Stable retained descriptors were mainly biomechanical, with a smaller recurrent PSI-derived component.

Figure 5 summarizes the cross-fold overlap structure of selected descriptors across the 5 development folds. A substantial shared overlap, together with several additional higher-overlap intersections, indicates that the retained descriptor set was not purely fold-specific but contained a reproducible core. Importantly, the higher-overlap intersections were dominated by biomechanical descriptors, supporting conventional biomechanics as the primary anchor of the stable feature set. PSI-derived descriptors appeared less frequently and in smaller numbers, but they were still recurrent across multiple higher-overlap intersections, indicating a complementary yet reproducible coordination-level contribution. Lower-overlap intersections were also present, suggesting some sensitivity of the selected panel to sample composition, although these intersections were generally smaller and more heterogeneous. Importantly, these stable descriptors were used for interpretation rather than as a fixed final feature panel. For final model construction, Fisher score ranking was reapplied once to the full development set, and the locked top-50 panel was selected from all candidate descriptors using all 72 development participants.

To further illustrate the main discriminative signals within this reproducible subset, Table 2 highlights representative descriptors that were both relatively stable across folds and highly ranked by Fisher score.

The representative examples make the reproducible structure more concrete and highlight two complementary, clinically meaningful patterns. First, the biomechanical examples are concentrated in interpretable domains, such as asymmetry, CoP, and gait timing, reinforcing conventional biomechanics as the primary anchor of the stable signal. This pattern is consistent with previous evidence that knee osteoarthritis is associated with abnormal plantar-pressure distribution, altered load redistribution, and CoP-related gait changes [6,16-18]. Second, PSI-derived descriptors were also retained among the stable high-ranking features, indicating that coordination-level information was recurrently represented alongside conventional biomechanical descriptors. Taken together, these representative descriptors indicate that ROA-related discrimination in the present cohort was anchored in clinically interpretable abnormalities of plantar loading, load redistribution, and whole-foot progression.

**Table 2.** Representative high-ranking and relatively stable subject-level descriptors selected from the reproducible subset across development folds. Selection rate denotes the proportion of development folds in which a descriptor was retained, and mean fold-wise Fisher score summarizes its average discriminative ranking within folds.

Descriptor	Selection rate	Fisher score, mean (SD)
asymmetry_std_sensor_5_std	1.0	7.92 (1.59)
asymmetry_mean_sensor_2_std	1.0	6.01 (3.82)
PSI^a^-Eigen-S-Min	1.0	5.62 (2.28)
PSI-Mean-S-Min	1.0	4.20 (1.70)
cop_variability_min	0.8	3.65 (0.42)
gait_timing_min	1.0	3.63 (1.46)
PSI-Edge-L-Std	0.6	3.17 (1.54)
PSI-Edge-L-Min	0.6	3.11 (0.60)

a PSI: Plantar Synergy Index.

### Phase-Resolved PSI Patterns Across Lag Scales and Functional Modules

To further interpret the coordination-level information captured by PSI, we examined whether ROA-related PSI differences showed coherent organization across lag scales, stance phase, and functional plantar modules. Because PSI was designed to characterize interregional temporal coupling rather than local pressure magnitude, the analysis was structured as a progressive visualization from sensor-pair spatial organization to phase-resolved stance dynamics, and finally to module-level difference trajectories. This design allowed us to assess whether PSI captured a distributed coordination pattern associated with ROA rather than isolated or nonspecific correlation differences.

**Figure 6.**
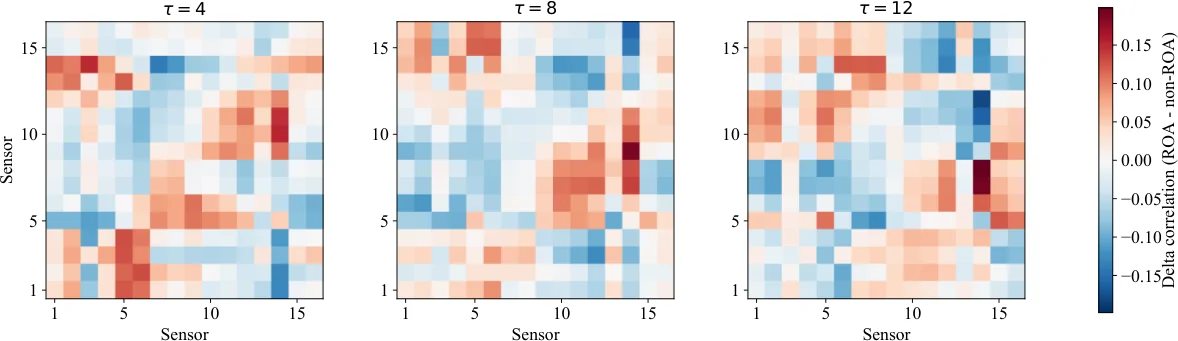
Sensor-pair delta-correlation heatmaps across representative lag settings. Heatmaps show the difference in lag-aligned sensor-pair correlation between ROA (radiographic knee osteoarthritis) and non-ROA groups (ROA minus non-ROA) at τ=4,8and12. Sensors 1‐8 and 9‐16 correspond to homologous plantar regions on the left and right feet, respectively. PSI (Plantar Synergy Index) differences were distributed across sensor pairs and varied by lag, rather than being confined to a single sensor region.

We first visualized sensor-pair delta-correlation matrices, defined as ROA minus non-ROA, at representative lag settings. As illustrated in Figure 6, the delta-correlation matrices revealed spatially distributed group differences across multiple sensor pairs, rather than a focal abnormality confined to a single plantar region. The pattern also varied across lag settings, indicating that ROA-related PSI alterations depended on the temporal offset used to characterize interregional coupling. At shorter lags, the observed differences may reflect altered near-synchronous load sharing among plantar regions, whereas longer lags may capture delayed coordination related to forward load transfer and bilateral stance progression. These findings suggest that PSI differences were not merely a consequence of local pressure magnitude changes, but reflected lag-dependent reorganization of plantar coordination.

**Figure 7.**
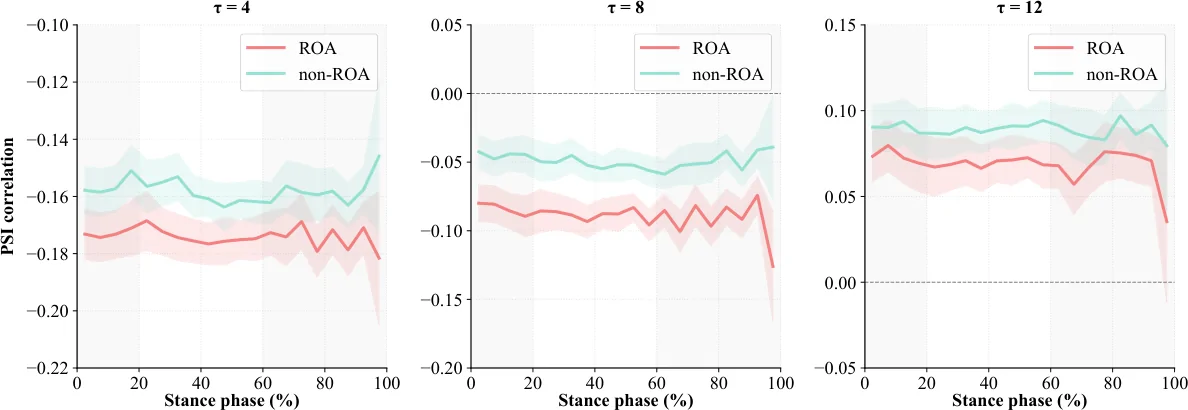
Phase-resolved PSI (Plantar Synergy Index) patterns for ROA (radiographic knee osteoarthritis) and non-ROA groups across lag scales. Stance phase was normalized to 0%‐100% and summarized in 5% bins. Curves represent group-level PSI summaries pooled across modules within each phase bin; shaded bands indicate 95% CIs. PSI differences emerged primarily in mid-to-late stance, consistent with altered load redistribution and propulsion-phase coordination in ROA.

The spatially distributed and lag-dependent delta-correlation patterns in Figure 6 suggested that ROA-related PSI differences were linked to the temporal organization of plantar coordination during walking. However, the heatmap representation does not indicate when during stance these coordination differences emerged. We therefore further aligned PSI trajectories to the normalized stance phase (0%‐100%) to examine the temporal expression of the observed lag-dependent coordination changes. As illustrated in Figure 7, group-level PSI trajectories differed between ROA and non-ROA across all examined lag settings. Across broad portions of stance, the ROA curves remained lower than the non-ROA curves, suggesting reduced or less coherent interregional plantar coordination in the ROA group. From a gait-phase perspective, this reduction was not limited to initial contact or early loading, but extended across mid-stance and late stance, where controlled CoP progression, forward load transfer, and preparation for push-off become increasingly important. The magnitude and temporal expression of this separation varied with lag scale; shorter lags emphasized more immediate interregional coupling, whereas longer lags showed greater fluctuation near late stance and push-off. These lag-dependent patterns suggest that the proposed PSI may capture ROA-related compensatory changes in plantar load redistribution, particularly during forward load transfer and propulsion, when coordinated forefoot and heel-to-midfoot interactions become biomechanically important.

**Figure 8.**
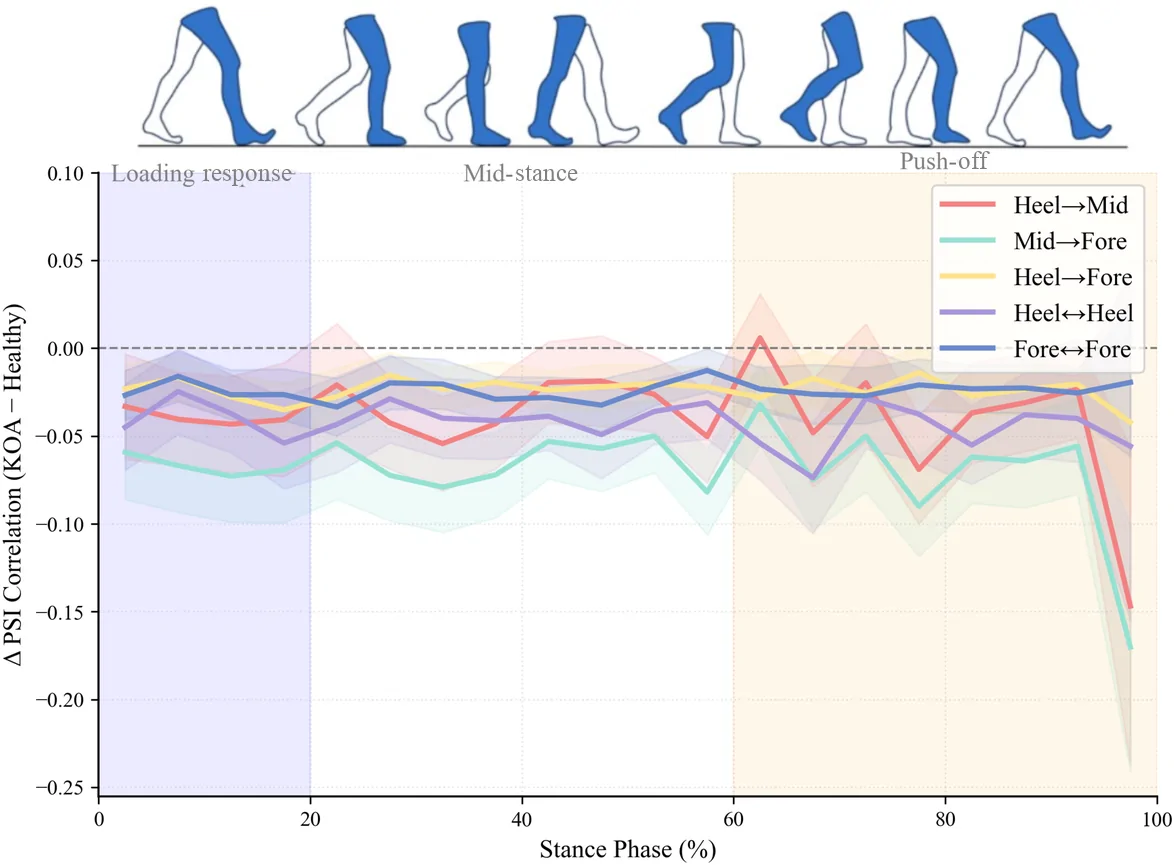
Phase-resolved PSI (Plantar Synergy Index) difference trajectories at the representative lag τ=0.4 s. Curves show $\Delta PSI=PSI_{ROA}-PSI_{non\text{-}ROA}$ for different inter-region functional modules across the normalized stance phase. Shaded background regions indicate major stance sub-phases. Cross-module PSI differences persisted across multiple stance-phase bins, indicating distributed inter-regional coupling changes rather than isolated within-module effects. ROA: radiographic knee osteoarthritis.

Figure 8 further shows phase-resolved PSI difference trajectories at a representative lag (τ=0.4s), where the vertical axis represents $\Delta PSI=PSI_{ROA}-PSI_{non\text{-}ROA}$. Across most of stance, the major module-level trajectories remained below zero, indicating that PSI was generally lower in ROA than in non-ROA for these interregion couplings. The persistence of negative differences across broad stance intervals suggests that ROA-related coordination changes were not restricted to a single gait event, but were expressed throughout stance-phase load transfer. This group difference persisted across broad portions of stance and was especially evident in forefoot-related and heel-to-mid couplings, while larger fluctuations near late stance and push-off suggested greater between-participant heterogeneity when forward load transfer and propulsion became dominant [6,16,17].

Taken together, Figures 6-8 indicate a coherent progression of PSI evidence: ROA-related differences were spatially distributed across sensor pairs, temporally expressed across the normalized stance phase, and functionally organized across interregion plantar modules. Importantly, these phase-resolved analyses were intended to characterize the organization of PSI differences, rather than to imply that all stance bins, lag scales, or modules contributed equally to the final classifier. The final retained descriptors were still determined by the participant-level feature-selection procedure.

### Robustness to Perturbation and Design Variation

We further examined whether PSI-related information remained useful under perturbation and design variation. Across temporal scaling, channel dropout, and Gaussian noise, fusion generally maintained the highest or among the highest participant-level *F*_1_-scores among the 3 models (Table 3). Performance was strongest at the native and mildly stretched temporal scales (1.0× and 1.2×, both *F*_1_-score=0.842), with only a modest reduction under compression (*F*_1_-score=0.824 at 0.8×). Under channel dropout and Gaussian noise, fusion also maintained a generally favorable profile relative to both PSI-only and bio-only models. These perturbation analyses suggest preliminary robustness of the combined representation under moderate signal variation, although the small locked test set limits strong comparative claims.

**Table 3.** Robustness of PSI-only, bio-only, and fusion models under temporal and signal perturbations. Reported values are subject-level *F*_1_-scores on the independent test set.

Setting and level	PSI^a^-only	Bio-only	Fusion
Temporal scaling			
0.8×	0.593	0.778	0.824
1.0×	0.609	0.778	0.842
1.2×	0.571	0.824	0.842
Channel dropout			
0.0	0.609	0.778	0.842
0.1	0.588	0.762	0.824
0.2	0.615	0.727	0.818
0.3	0.571	0.692	0.762
Gaussian noise			
0.00	0.609	0.778	0.842
0.01	0.600	0.778	0.824
0.03	0.571	0.727	0.800
0.05	0.556	0.667	0.818

a PSI: Plantar Synergy Index.

As shown in Table 4, across design variations, fusion showed *F*_1_-scores values within a relatively narrow range over sparsity thresholds (*F*_1_-score=0.824‐0.842) and lag settings (*F*_1_-score=0.818‐0.842), while showing greater sensitivity to window length (*F*_1_-score=0.761‐0.842). The 4-second window (80 samples at 20 Hz, spanning approximately 3‐4 complete gait cycles at typical walking cadences of 1.0‐1.2 Hz) showed the numerically highest *F*_1_-score among the examined window lengths. Shorter 3-second windows, covering only 1‐2 gait cycles, may have yielded less reliable correlation estimates for PSI matrix construction because the reduced sample size per window limited the statistical stability of the lag-aligned Pearson correlations. Conversely, 5-second windows, although providing more samples per correlation estimate, may have smoothed phase-specific coordination changes across stance subphases, reducing the ability to resolve temporally localized PSI differences between ROA and non-ROA groups. The 4-second window therefore appeared to provide a suitable balance between within-window statistical stability for correlation-based PSI estimation and temporal resolution across stance subphases.

Within the lag analysis, the multiscale configuration (T={4, 8, 12}, corresponding to 0.20, 0.40, and 0.60 s at 20 Hz) showed the numerically highest performance among the examined lag settings (*F*_1_-score=0.842), compared with each single-scale alternative. A single lag may not simultaneously capture rapid intrafoot pressure propagation between adjacent sensors and slower cross-region coordination reorganization spanning multiple functional modules. The multiscale design therefore provided a broader candidate coordination space—spanning short-to-intermediate coupling timescales relevant to mid-to-late stance gait dynamics—from which downstream Fisher score selection could retain informative lag-specific descriptors across all 3 scales. This exploratory pattern is consistent with the idea that the multiscale design may capture complementary coupling timescales, but it requires confirmation in larger independent cohorts. Overall, these exploratory perturbation and design-sensitivity analyses suggest that the selected configuration was not uniquely dependent on a single perturbation or design setting. These findings should be interpreted as preliminary robustness checks rather than as independent optimization or validation of the model design.

**Table 4.** Sensitivity of the fusion model to design variations. Reported values are participant-level *F*_1_-scores on the locked independent test set under alternative window lengths, lag configurations, and sparsity thresholds. These analyses assess robustness of the selected design rather than serving as final model tuning.

Setting and level	Fusion
Window length	
3 s (50%)	0.761
4 s (50%)	0.842
5 s (50%)	0.800
Lag configuration	
Single-*τ*=4	0.824
Single-*τ*=8	0.818
Single-*τ*=12	0.824
Multiscale	0.842
Sparsity threshold	
*θ*=0.01	0.824
*θ*=0.02	0.842
*θ*=0.05	0.842

### Benchmarking Against Representative Convolutional Neural Network, Transformer, and Graph-Based Baselines

Table 5 shows that fusion achieved the strongest overall participant-level performance among the evaluated models. Among the baselines, InceptionTime [31] was the strongest competitor and showed a relatively balanced classification profile, but remained below fusion in accuracy, *F*_1_-score, AUROC, precision, and recall, while matching the best specificity. Medformer [32] achieved high recall comparable with fusion, but with lower AUROC, lower precision, and markedly reduced specificity, indicating a more false-positive−prone decision profile. TodyNet [33] showed perfect recall but near-zero specificity, yielding the weakest overall discrimination and suggesting a strong bias toward positive predictions under the present data scale.

Overall, these results suggest that, under the current data scale and evaluation setting, fusion showed a directionally favorable threshold-dependent profile relative to the evaluated end-to-end baselines. Relative to InceptionTime, the strongest baseline, fusion showed numerically higher accuracy, *F*_1_-score, AUROC, precision, and recall while matching specificity, but these differences should be interpreted cautiously given the small locked test set. Medformer preserved high recall but at the cost of lower specificity and lower overall discrimination, whereas TodyNet showed a marked false-positive tendency with poor ranking quality. Together, these comparisons suggest that the proposed representation-based framework remained competitive with representative end-to-end baselines in this proof-of-concept evaluation.

**Table 5.** Participant-level comparison between the proposed fusion model and representative end-to-end baselines on the independent test set. All models used the same participant-wise development/test split, fixed-length input, and participant-level evaluation protocol.

Model	Accuracy	*F*_1_-score	AUROC^a^	Precision	Recall	Specificity
InceptionTime	0.800	0.778	0.802	0.778	0.778	0.818
Medformer	0.700	0.727	0.707	0.615	0.889	0.545
TodyNet	0.450	0.621	0.485	0.450	1.000	0.000
Fusion	0.850	0.842	0.818	0.800	0.889	0.818

a AUROC: area under the receiver operating characteristic curve.

## Discussion

### Principal Results

This study addressed a key representation gap in plantar-pressure–based ROA identification by introducing a coordination-aware framework that integrates local biomechanical loading with multiscale interregional temporal information. The combined framework achieved competitive participant-level ROA identification, but it did not show consistent superiority over bio-only across performance metrics. The biomechanics branch made the strongest individual contribution, whereas PSI showed limited standalone discrimination. However, PSI captured complementary coordination patterns that were not represented by conventional biomechanical descriptors, and a reproducible PSI component was retained within the combined representation. Robustness and design-sensitivity analyses provided preliminary evidence that the findings were not confined to a single evaluated configuration. Accordingly, the principal innovation of this study is representational and methodological rather than cohort- or device-based; it explicitly combines interpretable local loading descriptors with multiscale interregional temporal coordination under a participant-level, leakage-controlled evaluation protocol.

The branch-wise findings help explain the primary source of discrimination. The strong contribution of the biomechanics branch is consistent with previous evidence that ROA-related plantar-pressure abnormalities occur in anatomically meaningful regions, particularly the hallux and heel, and are also reflected in CoP stability and variability [17,18]. This interpretation is further supported by recent studies associating ROA-related functional impairment with altered plantar-pressure behavior, gastrocnemius muscle-tendon-unit stiffness, and 3D gait biomechanics [7,8]. These patterns are biomechanically plausible because structural degeneration, pain-related gait adaptation, and altered neuromuscular control at the knee may be associated with compensatory load redistribution and changes in stance progression [6-8,15-18]. Experimental modulation of tibialis anterior activity has also been shown to alter plantar pressure and gastrocnemius activity during gait, supporting the sensitivity of plantar loading to neuromuscular modulation [37]. The findings of this study therefore reinforce the importance of anatomically grounded loading descriptors for participant-level ROA identification.

The phase-resolved and fold-wise analyses help clarify what PSI may contribute beyond these local loading descriptors. The observed PSI differences were distributed across multiple functional modules and lag settings rather than being confined to isolated local extrema. In addition, a core group of PSI descriptors was repeatedly retained across the development folds, supporting the reproducibility of these selected patterns within the present dataset in accordance with established feature-stability principles [34,35]. Although PSI is not a direct measure of joint kinematics or electromyographic muscle activity, its distributed temporal patterns are compatible with studies reporting altered kinematic synergy, muscle synergy, and muscle-activation patterns in individuals with KOA during walking [38-40]. PSI may therefore provide an interpretable coordination-level description of bilateral load transfer and whole-foot progression. However, feature stability and phase-resolved interpretability do not by themselves establish incremental predictive value beyond the biomechanics branch. Given the limited standalone discrimination of PSI and the absence of consistent superiority of fusion over bio-only, the findings of this study should not be interpreted as definitive evidence of additional predictive or clinical value.

Comparisons with the end-to-end baselines place these findings in a broader modeling context. The evaluated models represented complementary learning paradigms but exhibited different operating profiles: InceptionTime was the strongest and most balanced competitor, Medformer favored sensitivity over specificity, and TodyNet showed lower specificity and a higher false-positive tendency in the present locked test set. The value of the proposed framework therefore lies less in definitive performance superiority over every alternative than in combining competitive participant-level identification with an explicit and interpretable organization of plantar-pressure information before classification. The robustness and design-sensitivity analyses further suggested that the main findings were not entirely dependent on a single analytical configuration, although these analyses remain internal and exploratory. Together with the structured comparison in Multimedia Appendix 5, these results support the positioning of the framework as a representational and methodological contribution rather than a cohort- or device-based advance.

To provide a screening-context interpretation of the locked-test operating characteristics, we translated sensitivity and specificity into positive predictive value and negative predictive value across assumed community ROA prevalence levels in Multimedia Appendix 8 [41]. This supplementary analysis illustrates how disease prevalence could affect the potential rule-in and rule-out value of the framework. It should not, however, be interpreted as evidence of clinical deployment readiness because the underlying operating characteristics were estimated from a small internal test set rather than from a representative prospective screening population.

### Limitations

Several limitations should be acknowledged. The sample size was modest, and all participants were drawn from a single previously reported cohort, limiting the precision and external generalizability of the performance estimates. Although the locked test set was separated from model development, it remained part of the same source cohort and therefore represents an internal held-out proof-of-concept evaluation rather than external validation. Contemporary prediction-model guidance emphasizes that independent validation should be adequately powered to estimate performance with sufficient precision [14,42]. The findings of this study should consequently be interpreted as preliminary and require confirmation in larger independent cohorts.

Important clinical and demographic factors, including age, BMI, and affected-side heterogeneity, may influence plantar-pressure patterns [15]. The supplementary covariate sensitivity analysis indicated that the available demographic variables alone did not reproduce the performance of the fusion model and that their inclusion did not materially improve its performance (Multimedia Appendix 6). Nevertheless, this analysis cannot exclude residual confounding or establish the independent and interactive effects of age and BMI in the present modest sample. These questions will require larger, more carefully stratified cohorts with richer clinical covariate information. Furthermore, to our knowledge, no publicly available dataset combined confirmed ROA labels with suitable in-shoe plantar-pressure recordings for direct external validation. Available public resources lacked either ROA annotations or compatible pressure measurements, precluding cross-dataset benchmarking [43,44]. Multicenter efforts to establish open datasets with imaging-grade clinical labels and standardized plantar-pressure recordings would substantially strengthen future evaluation.

This study also focused on binary ROA identification rather than disease-severity stratification or longitudinal progression. PSI depends on sensor layout, signal quality, and preprocessing choices, which may affect the validity and comparability of plantar-pressure measurements [10]; its transferability across devices and clinical settings therefore remains uncertain. Recent Soft Science studies demonstrate advances in biocompatible piezoelectric, gel-based, and electrospun flexible pressure sensors for walking, human-motion, gait, and continuous health monitoring [45-47]. These developments strengthen the technological basis for wearable plantar sensing, but they also underscore the need to verify signal stability, durability, environmental resilience, and cross-platform calibration before clinical translation [45-47]. In addition, the proposed coordination-level interpretation was not directly validated using concurrent joint-kinematic, electromyographic, or other neuromuscular measurements. Future studies should validate the framework prospectively in larger multicenter cohorts, evaluate its robustness across sensor platforms and patient subgroups, examine its association with direct biomechanical and neuromuscular measurements, and extend it toward severity modeling and longitudinal prediction.

### Conclusions

This study introduces an interpretable participant-level representation that integrates local biomechanical loading with multiscale interregional temporal coordination, extending plantar-pressure approaches centered primarily on summary descriptors or less interpretable end-to-end models. Complementary evaluations provided convergent evidence of competitive participant-level ROA identification and preliminary robustness across the evaluated settings, while showing that local biomechanical descriptors constituted the main discriminative basis and that PSI offered a reproducible coordination-level component whose incremental predictive value remains to be established. By broadening plantar-pressure analysis from local loading to distributed gait coordination, the framework provides a methodological basis for more interpretable functional characterization of ROA. If prospectively validated in external multicenter cohorts, it could warrant further evaluation as an adjunct to imaging for functional assessment and longitudinal monitoring. Prospective multicenter validation is required before clinical translation.

## Supplementary material

10.2196/101026 Multimedia Appendix 1 Participant flow and baseline characteristics.

10.2196/101026 Multimedia Appendix 2 Sensor-to-anatomy mapping and biomechanical feature definitions.

10.2196/101026 Multimedia Appendix 3 Explicit forms of Plantar Synergy Index.

10.2196/101026 Multimedia Appendix 4 Implementation and training hyperparameters.

10.2196/101026 Multimedia Appendix 5 Cross-literature comparison.

10.2196/101026 Multimedia Appendix 6 Covariate sensitivity analysis.

10.2196/101026 Multimedia Appendix 7 Bootstrap confidence intervals and DeLong comparisons.

10.2196/101026 Multimedia Appendix 8 Predictive values across community prevalence levels.

## Abbreviations

AUROC: area under the receiver operating characteristic curve
CoP: center of pressure
IRB: Institutional Review Board
JARS: *Journal Article Reporting Standards*
K-L: Kellgren-Lawrence
MLP: multilayer perceptron
PROC: Pearl River Osteoarthritis Cohort
PSI: Plantar Synergy Index
RF: random forest
ROA: radiographic knee osteoarthritis
