# Supplementary material for: Radiographic Knee Osteoarthritis Identification Using Wearable Plantar Pressure: Secondary Cross-Sectional Analysis of Interpretable Framework Integrating Biomechanical Descriptors and the Multiscale Synergy Index
Source: JMIR Mhealth Uhealth. 2026 Sep 11;14:e101026. doi: 10.2196/101026 (PMC13566073; doi:10.2196/101026)
Supplement: Supplementary file 1 [file mhealth-v14-e101026-s001.docx]

Figure S1 presents the JARS Participant Flowchart documenting participant flow from cohort enrollment through the final analytic sample. Baseline demographic and anthropometric characteristics of the 92-participant plantar-pressure cohort, stratified by radiographic knee osteoarthritis (ROA) status, are summarized in Table 1.

Figure S1. JARS Participant Flowchart for the 92-participant plantar-pressure cohort from the Pearl River Osteoarthritis Cohort (PROC), Zhujiang Hospital, Guangzhou.

Table S1. Baseline demographic and anthropometric characteristics stratified by ROA status in the 92-participant plantar-pressure cohort. Normality was assessed within each group using Shapiro-Wilk tests; Welch's t-test was used when both groups were approximately normal, and Mann-Whitney U tests were used otherwise. Cohen’s d is reported with positive values indicating higher values in the non-ROA group.

| Variable | non-ROA | ROA | Test | P-value | Cohen’s d |
| --- | --- | --- | --- | --- | --- |
| Age (years) | 59.76 ± 7.93 | 66.58 ± 7.45 | Mann-Whitney | <.001 | -0.885 |
| Height (cm) | 159.31 ± 7.14 | 156.67 ± 8.45 | Mann-Whitney | .10 | 0.338 |
| Weight (kg) | 60.17 ± 9.14 | 62.65 ± 11.78 | Welch t-test | .27 | -0.237 |
| BMI (kg/m²) | 23.72 ± 3.08 | 25.40 ± 3.63 | Welch t-test | .02 | -0.504 |

The ROA group was significantly older (P < .001) and had higher BMI (P = .02), consistent with the known epidemiology of knee osteoarthritis. Individual-level sex and K-L grade data were not available in the current analysis dataset; group-level summaries have been reported in the original studies.
