# Supplementary material for: Radiographic Knee Osteoarthritis Identification Using Wearable Plantar Pressure: Secondary Cross-Sectional Analysis of Interpretable Framework Integrating Biomechanical Descriptors and the Multiscale Synergy Index
Source: JMIR Mhealth Uhealth. 2026 Sep 11;14:e101026. doi: 10.2196/101026 (PMC13566073; doi:10.2196/101026)
Supplement: Supplementary file 2 [file mhealth-v14-e101026-s002.docx]

This appendix provides: (i) the 16-channel sensor-to-anatomy mapping used to group plantar-pressure sensors into forefoot, midfoot, and hindfoot regions (Table 1), and (ii) the explicit definitions and computational formulas for the 35 window-level biomechanical features extracted from raw plantar-pressure windows (features g₀–g₃₄).

Table S1. Sensor index to anatomical region mapping for the 16-channel in-shoe plantar-pressure system.

| Sensor Index (Left / Right) | Foot Region | Anatomical Landmark |
| --- | --- | --- |
| 1_L / 1_R | Forefoot | Great toe |
| 2_L / 2_R | Forefoot | First metatarsal head (MTH1) |
| 3_L / 3_R | Forefoot | Second–third metatarsal heads (MTH23) |
| 4_L / 4_R | Forefoot | Fourth–fifth metatarsal heads (MTH45) |
| 5_L / 5_R | Midfoot | Medial midfoot |
| 6_L / 6_R | Midfoot | Lateral midfoot |
| 7_L / 7_R | Hindfoot | Medial heel |
| 8_L / 8_R | Hindfoot | Lateral heel |

All biomechanical features in this appendix were computed from raw plantar-pressure windows. For notational simplicity, $P(t,s)$ denotes the raw plantar-pressure value at time $t$ and sensor $s$ within a window, rather than the z-scored signal used in the PSI branch.

## Basic statistical features (10 features, g0–g9)

For left-foot samples $\{P(t,s):t=1..L,s\mathcal{\in L\}}$:

$$\begin{aligned} g_{0}=\frac{1}{8L}\sum_{t=1}^{L} \sum_{s\mathcal{\in L}} P\left( t,s \right)\#\left( 1 \right) \end{aligned}$$

$$\begin{aligned} g_{1}=\sqrt{\frac{1}{8L}\sum_{t=1}^{L} \sum_{s\mathcal{\in L}} (P\left( t,s \right)-g_{0})^{2}}\#(2) \end{aligned}$$

$$\begin{aligned} g_{2}=\max_{t,s\mathcal{\in L}}P\left( t,s \right)\#\left( 3 \right) \end{aligned}$$

$$\begin{aligned} g_{3}=\min_{t,s\mathcal{\in L}}P\left( t,s \right)\#\left( 4 \right) \end{aligned}$$

$$\begin{aligned} g_{4}=median\left( \{P(t,s){\}}_{t=1..L,s\mathcal{\in L}} \right)\#\left( 5 \right) \end{aligned}$$

Right-foot counterparts define $g_{5}$–$g_{9}$ analogously.

## Left-right asymmetry features (16 features, g10–g25)

For each paired channel $(s,\pi(s))$, $s=1,\ldots,8$:

$$\begin{aligned} \bar{P}_{L}\left( s \right)=\frac{1}{L}\sum_{t=1}^{L} P\left( t,s \right)\#\left( 6 \right) \end{aligned}$$

$$\begin{aligned} \bar{P}_{R}\left( s \right)=\frac{1}{L}\sum_{t=1}^{L} P\left( t,\pi\left( s \right) \right)\#\left( 7 \right) \end{aligned}$$

$$\begin{aligned} g_{9+s}=\left| \bar{P}_{L}\left( s \right)-\bar{P}_{R}\left( s \right) \right|,s=1,\ldots,8\#\left( 8 \right) \end{aligned}$$

Define per-channel temporal standard deviation:

$$\begin{aligned} \sigma_{L}\left( s \right)=\sqrt{\frac{1}{L}\sum_{t=1}^{L} (P\left( t,s \right)-\bar{P}_{L}\left( s \right))^{2}}\#\left( 9 \right) \end{aligned}$$

$$\begin{aligned} \sigma_{R}\left( s \right)=\sqrt{\frac{1}{L}\sum_{t=1}^{L} (P\left( t,\pi\left( s \right) \right)-\bar{P}_{R}\left( s \right))^{2}}\#\left( 10 \right) \end{aligned}$$

$$\begin{aligned} g_{17+s}=\left| \sigma_{L}\left( s \right)-\sigma_{R}\left( s \right) \right|,s=1,\ldots,8\#\left( 11 \right) \end{aligned}$$

## Load distribution features (4 features, g26–g29)

Define instantaneous total load per foot:

$$\begin{aligned} L_{\mathrm{total}}\left( t \right)=\sum_{s\mathcal{\in L}} P\left( t,s \right),R_{\mathrm{total}}\left( t \right)=\sum_{s\mathcal{\in R}} P\left( t,s \right)\#\left( 12 \right) \end{aligned}$$

$$\begin{aligned} \rho\left( t \right)=\frac{L_{\mathrm{total}}\left( t \right)}{R_{\mathrm{total}}\left( t \right)+\epsilon},\epsilon={10}^{-8}\#\left( 13 \right) \end{aligned}$$

Then

$$\begin{aligned} g_{26}=\frac{1}{L}\sum_{t=1}^{L} \rho\left( t \right)\#\left( 14 \right) \end{aligned}$$

$$\begin{aligned} g_{27}=\sqrt{\frac{1}{L}\sum_{t=1}^{L} (\rho\left( t \right)-g_{26})^{2}}\#\left( 15 \right) \end{aligned}$$

$$\begin{aligned} g_{28}=\max_{t}\rho\left( t \right)\#\left( 16 \right) \end{aligned}$$

$$\begin{aligned} g_{29}=\min_{t}\rho\left( t \right)\#\left( 17 \right) \end{aligned}$$

## Temporal asymmetry feature (1 feature, g30)

Define peak-time index in each paired channel:

$$\begin{aligned} t_{\mathrm{peak}}^{L}\left( s \right)=arg\max_{1\leq t\leq L}P\left( t,s \right),t_{\mathrm{peak}}^{R}\left( s \right)=arg\max_{1\leq t\leq L}P\left( t,\pi\left( s \right) \right)\#\left( 18 \right) \end{aligned}$$

$$\begin{aligned} g_{30}=\left| \frac{1}{8}\sum_{s=1}^{8} t_{\mathrm{peak}}^{L}\left( s \right)-\frac{1}{8}\sum_{s=1}^{8} t_{\mathrm{peak}}^{R}\left( s \right) \right|\#\left( 19 \right) \end{aligned}$$

## Regional pressure features (2 features, g31–g32)

Define region channel sets (bilateral merged):

$$\begin{aligned} \mathcal{F}_{\mathrm{med}}=1,2,3,4,9,10,11,12\mathcal{,M=}5,6,13,14\mathcal{,H=}7,8,15,16\#\left( 20 \right) \end{aligned}$$

Define region-mean pressure at each time point:

$$\begin{aligned} P_{\mathcal{G}}\left( t \right)=\frac{1}{\left| \mathcal{G} \right|}\sum_{s\in\mathcal{G}} P\left( t,s \right),\mathcal{G}\in\mathcal{F}_{\mathrm{med}}\mathcal{,M,H\#}\left( 21 \right) \end{aligned}$$

with temporal mean $\overline{P}_{\mathcal{G}}=\frac{1}{L}\sum_{t=1}^{L} P_{\mathcal{G}}(t)$. Then (with $\epsilon={10}^{-8}$):

$$\begin{aligned} g_{31}=\frac{\overline{P}_{\mathcal{F}_{\mathrm{med}}}}{\overline{P}_{\mathcal{H}}+\epsilon}\#\left( 22 \right) \end{aligned}$$

$$\begin{aligned} g_{32}=\frac{\overline{P}_{\mathcal{M}}}{\overline{P_{\mathcal{F}_{\mathrm{med}}}}+\overline{P_{\mathcal{H}}}+\epsilon}\#\left( 23 \right) \end{aligned}$$

## Gait-timing feature (1 feature, g33)

Define regional peak time:

$$\begin{aligned} t_{\mathcal{F}}=arg\max_{1\leq t\leq L}P_{\mathcal{F}_{\mathrm{med}}}\left( t \right),t_{\mathcal{H}}=arg\max_{1\leq t\leq L}P_{\mathcal{H}}\left( t \right)\#\left( 24 \right) \end{aligned}$$

$$\begin{aligned} g_{33}=\left| t_{\mathcal{H}}-t_{\mathcal{F}} \right|\#\left( 25 \right) \end{aligned}$$

## Center-of-pressure feature (1 feature, g34)

Let instantaneous total pressure be $S(t)=\sum_{s=1}^{16} P(t,s)$. Using channel index as discrete lateral coordinate:

$$\begin{aligned} \mathrm{CoP}_{x}\left( t \right)=\frac{\sum_{s=1}^{16} (s-1)P(t,s)}{S\left( t \right)+\epsilon},\epsilon={10}^{-8}\#\left( 26 \right) \end{aligned}$$

Define temporal variance (or equivalent standard deviation) as CoP stability feature:

$$\begin{aligned} {\overline{\mathrm{CoP}}}_{x}=\frac{1}{L}\sum_{t=1}^{L} \mathrm{CoP}_{x}\left( t \right),g_{34}=\frac{1}{L}\sum_{t=1}^{L} \left( \mathrm{CoP}_{x}\left( t \right)-{\overline{\mathrm{CoP}}}_{x} \right)^{2}\#\left( 27 \right) \end{aligned}$$

## 35-D window-level biomechanical feature vector

The final window-level biomechanical feature vector is

$$\begin{aligned} \boldsymbol{g}^{\left( w \right)}=\left[ g_{0}^{\left( w \right)},g_{1}^{\left( w \right)},\cdots,g_{34}^{\left( w \right)} \right]^{\top}\in\mathbb{R}^{35}\#\left( 28 \right) \end{aligned}$$

Its grouped components are: basic statistics ($g_{0}$–$g_{9}$), left-right asymmetry ($g_{10}$–$g_{25}$), load distribution ($g_{26}$–$g_{29}$), temporal asymmetry ($g_{30}$), regional pressure ($g_{31}$–$g_{32}$), gait timing ($g_{33}$), and CoP feature ($g_{34}$).
