# Supplementary material for: Radiographic Knee Osteoarthritis Identification Using Wearable Plantar Pressure: Secondary Cross-Sectional Analysis of Interpretable Framework Integrating Biomechanical Descriptors and the Multiscale Synergy Index
Source: JMIR Mhealth Uhealth. 2026 Sep 11;14:e101026. doi: 10.2196/101026 (PMC13566073; doi:10.2196/101026)
Supplement: Supplementary file 3 [file mhealth-v14-e101026-s003.docx]

This appendix provides the explicit forms of the PSI-branch operations and statistics involved in Eqs. (2)–(4). The main text keeps only key definitions and the pipeline.

## Density statistics: indicator matrix and effective edge count

Given threshold $\theta$, define

$$\begin{aligned} A_{ij}^{\left( w \right)}\left( \tau\right)=\mathbb{I}\left( C_{ij}^{\left( w \right)}\left( \tau\right)\geq\theta\right)\#\left( 1 \right) \end{aligned}$$

and the effective edge count

$$\begin{aligned} f_{\mathrm{edge}}^{\left( w \right)}\left( \tau\right)=\sum_{i=1}^{C} \sum_{j=1}^{C} A_{ij}^{\left( w \right)}\left( \tau\right)\#\left( 2 \right) \end{aligned}$$

Here, $\theta$ is only used as the counting threshold for density statistics. Other summaries are computed directly from real-valued $C^{(w)}(\tau)$.

## Synergy-strength summaries on the off-diagonal set

Let the off-diagonal index set be $\Omega=\{(i,j)\mid i\neq j\}$ with cardinality $|\Omega|=C(C-1)$. Define

$$\begin{aligned} f_{\mathrm{mean}}^{\left( w \right)}\left( \tau\right)=\frac{1}{\left| \Omega\right|}\sum_{\left( i,j \right)\in\Omega} C_{ij}^{\left( w \right)}\left( \tau\right)\#\left( 3 \right) \end{aligned}$$

$$\begin{aligned} f_{\mathrm{std}}^{\left( w \right)}\left( \tau\right)=\sqrt{\frac{1}{\left| \Omega\right|}\sum_{\left( i,j \right)\in\Omega} \left( C_{ij}^{\left( w \right)}\left( \tau\right)-f_{\mathrm{mean}}^{\left( w \right)}\left( \tau\right) \right)^{2}}\#\left( 4 \right) \end{aligned}$$

Extrema summaries are

$$\begin{aligned} f_{\max}^{\left( w \right)}\left( \tau\right)=\max_{\left( i,j \right)\in\Omega}C_{ij}^{\left( w \right)}\left( \tau\right)\#\left( 5 \right) \end{aligned}$$

$$\begin{aligned} f_{\min}^{\left( w \right)}\left( \tau\right)=\min_{\left( i,j \right)\in\Omega}C_{ij}^{\left( w \right)}\left( \tau\right)\#\left( 6 \right) \end{aligned}$$

## Dominant spectral mode strength

The leading-eigenvalue statistic is

$$\begin{aligned} \tilde{C}^{\left( w \right)}\left( \tau\right)=\frac{C^{\left( w \right)}\left( \tau\right)+\left( C^{\left( w \right)}\left( \tau\right) \right)^{\top}}{2}\#\left( 7 \right) \end{aligned}$$

$$\begin{aligned} f_{\lambda}^{\left( w \right)}\left( \tau\right)=\lambda_{\max}\left( \tilde{C}^{\left( w \right)}\left( \tau\right) \right)\#\left( 8 \right) \end{aligned}$$

## Lagged coupling asymmetry statistic

The asymmetry statistic is

$$\begin{aligned} f_{\mathrm{asym}}^{\left( w \right)}\left( \tau\right)=\frac{1}{C^{2}}\sum_{i=1}^{C} \sum_{j=1}^{C} \left| C_{ij}^{\left( w \right)}\left( \tau\right)-C_{ji}^{\left( w \right)}\left( \tau\right) \right|\#\left( 9 \right) \end{aligned}$$

## 7-D summary vector and 21-D concatenation

For each lag scale $\tau$, the 7-D summary is

$$\boldsymbol{f}_{\mathrm{PSI}}^{\left( w \right)}(\tau)=[f_{\mathrm{edge}}^{\left( w \right)}(\tau),f_{\mathrm{mean}}^{\left( w \right)}(\tau),f_{\mathrm{std}}^{\left( w \right)}(\tau),f_{\max}^{\left( w \right)}(\tau),f_{\min}^{\left( w \right)}(\tau),f_{\lambda}^{\left( w \right)}(\tau),f_{\mathrm{asym}}^{\left( w \right)}(\tau)]^{\top}()$$

Concatenating over $\tau\in\{4,8,12\}$ gives the 21-D window-level PSI feature:

$$\begin{aligned} f_{\mathrm{PSI}}^{\left( w \right)}=\left[ \boldsymbol{f}_{\mathrm{PSI}}^{\left( w \right)}\left( 4 \right);\boldsymbol{f}_{\mathrm{PSI}}^{\left( w \right)}\left( 8 \right);\boldsymbol{f}_{\mathrm{PSI}}^{\left( w \right)}\left( 12 \right) \right]\in\mathbb{R}^{21}\#\left( 10 \right) \end{aligned}$$

## Compact naming convention for retained PSI descriptors

For presentation clarity, retained PSI-derived descriptors are denoted using a compact naming scheme. Each retained feature name contains: (i) the PSI statistic type, (ii) the lag-scale label, and (iii) the subject-level aggregation suffix. Scale labels S, M, and L correspond to τ = 4, 8, and 12, respectively. Statistic types include Edge, Mean, Std, Max, Min, $\lambda$, and Asym, corresponding to the seven PSI features.
