# Supplementary material for: Radiographic Knee Osteoarthritis Identification Using Wearable Plantar Pressure: Secondary Cross-Sectional Analysis of Interpretable Framework Integrating Biomechanical Descriptors and the Multiscale Synergy Index
Source: JMIR Mhealth Uhealth. 2026 Sep 11;14:e101026. doi: 10.2196/101026 (PMC13566073; doi:10.2196/101026)
Supplement: Supplementary file 4 [file mhealth-v14-e101026-s004.docx]

This appendix lists the key implementation and training hyperparameters for the primary Random Forest model and the three representative end-to-end baselines. All models were evaluated under the same subject-wise development/test split (72/20 subjects), fixed-length input (80 samples × 16 channels), and subject-level evaluation protocol.

## Primary Model

Key implementation and training hyperparameters

| Module | Hyperparameter | Value |
| --- | --- | --- |
| Mixer / PSI encoder | MixerBlock layers | 2 |
|  | Hidden dimension | 32 |
|  | Dropout | 0.1 |
|  | Sequence length | 80 |
|  | Input channels | 16 |
|  | Optimizer | AdamW |
|  | Learning rate | 0.001 |
|  | Weight decay | 0.0001 |
|  | Max epochs | 80 |
|  | Early stopping patience | 12 |
|  | Loss function | MSE |
| Random Forest classifier | n_estimators | 800 |
|  | max_depth | 6 |
|  | min_samples_split | 4 |
|  | min_samples_leaf | 2 |
|  | class_weight | balanced |
|  | max_features | log2 |
|  | bootstrap | True |
| Development/test split | Development set size | 72 |
|  | Independent test set size | 20 |
| Threshold optimization | Threshold source | pooled OOF predictions |
|  | Search range | [0.10, 0.90] |
|  | Search step | 0.05 |
| Experimental setup | Optimization metric  Random Seed | max F1  3402 |

## End-to-End Baselines

Key model-specific implementation and training hyperparameters for the representative end-to-end baselines.

| Baseline | Hyperparameter | Value |
| --- | --- | --- |
| InceptionTime | Convolutional kernels | 9, 19, 39 |
|  | Number of filters | 32 |
|  | Optimizer | Adam |
|  | Learning rate | 0.001 |
|  | Weight decay | 0.0001 |
|  | Batch size | 32 |
|  | Max epochs | 100 |
|  | Early stopping patience | 20 |
|  | Validation schedule | Every 5 epochs |
|  | Class weighting | Fixed class weights: 1.0 / 2.0 |
|  | Model selection criterion | Validation subject-level $F1$ |
| Medformer | Patch sizes | 8, 16, 32 |
|  | Patch strides | 4, 8, 16 |
|  | Hidden dimension ($d_{\mathrm{model}}$) | 128 |
|  | Number of heads | 8 |
|  | Number of encoder layers | 4 |
|  | Feed-forward dimension ($d_{ff}$) | 256 |
|  | Dropout | 0.1 |
|  | Optimizer | AdamW |
|  | Learning rate | 0.0003 |
|  | Weight decay | 0.0001 |
|  | Batch size | 32 |
|  | Max epochs | 100 |
|  | Early stopping patience | 12 |
|  | Validation schedule | Every 5 epochs |
|  | Class weighting | Fold-specific class weights |
|  | Model selection criterion | Validation subject-level $F1$ |
| TodyNet | Hidden dimension | 128 |
|  | Number of graph blocks | 3 |
|  | Top-$k$ neighbors | 8 |
|  | Temporal kernel sizes | 7, 5 |
|  | Dropout | 0.1 |
|  | Optimizer | AdamW |
|  | Learning rate | 0.0005 |
|  | Weight decay | 0.0001 |
|  | Batch size | 32 |
|  | Max epochs | 100 |
|  | Early stopping patience | 12 |
|  | Validation schedule | Every 5 epochs |
|  | Class weighting | Fold-specific class weights |
|  | Model selection criterion | Validation subject-level $F1$ |
