# Supplementary material for: Radiographic Knee Osteoarthritis Identification Using Wearable Plantar Pressure: Secondary Cross-Sectional Analysis of Interpretable Framework Integrating Biomechanical Descriptors and the Multiscale Synergy Index
Source: JMIR Mhealth Uhealth. 2026 Sep 11;14:e101026. doi: 10.2196/101026 (PMC13566073; doi:10.2196/101026)
Supplement: Supplementary file 5 [file mhealth-v14-e101026-s005.docx]

This appendix provides a structured comparison of the present framework with prior plantar-pressure and gait-based studies along key dimensions: modality, feature type, coordination modeling, evaluation level, leakage control, and reported metric.

Prior work on the same cohort used handcrafted biomechanical features exclusively, evaluated at the window level, and did not explicitly report leakage-controlled subject-level metrics or coordination-level features. A prior insole-based gait phenotyping study used a different cohort, device, and outcome definition. The present framework differs from prior work primarily in feature representation, coordination modeling, evaluation level, and subject-wise leakage control, while using the same cohort and plantar-pressure modality as prior cohort studies.

Table S1. Cross-literature comparison along key dimensions: feature type, evaluation protocol, leakage control, and interpretability.

| **Dimension** | **Prior plantar-pressure classification study [13]** | **Prior plantar-pressure functional-monitoring study [14]** | **Prior insole-based gait phenotyping study [11]** | **This Study** |
| --- | --- | --- | --- | --- |
| Modality | Plantar-pressure | Plantar-pressure | Insole kinetics | Plantar-pressure |
| Cohort | Same N=92 | Same N=92 | 60 (22+38, different) | Same N=92 |
| Feature Type | Handcrafted biomech only | Multidimensional biomech | Gait signatures (vGRF) | Biomech + PSI coordination |
| Evaluation Level | Window-level (classification) | Subject-level (regression) | Subject-level | Subject-level (classification) |
| Leakage Control | Not explicit | Not explicit | Not reported | Strict subject-wise splitting |
| Reported Metric | Acc = 82.61%, F1 = 0.800 (AUC not reported) | MAE = 2.69 s (40mFPWT), MAPE = 9.6% (regression; no Acc/F1/AUC) | auROC = 0.83 (insole test set, controls vs knee OA) | F1 = 0.842, AUC = 0.818 |
