# Supplementary material for: Radiographic Knee Osteoarthritis Identification Using Wearable Plantar Pressure: Secondary Cross-Sectional Analysis of Interpretable Framework Integrating Biomechanical Descriptors and the Multiscale Synergy Index
Source: JMIR Mhealth Uhealth. 2026 Sep 11;14:e101026. doi: 10.2196/101026 (PMC13566073; doi:10.2196/101026)
Supplement: Supplementary file 6 [file mhealth-v14-e101026-s006.docx]

This appendix provides a two-layer model-level covariate sensitivity analysis to assess whether the observed classification performance could be explained primarily by demographic covariates (Age, Height, Weight, BMI) rather than plantar-pressure signal features.

First, a demographics-only Random Forest classifier using Age, Height, Weight, and BMI was trained and evaluated under the same subject-wise protocol as the primary models. Second, Age, Height, Weight, and BMI were concatenated with the Fusion feature set before Fisher-score selection and RF classification. The demographic variables' Fisher F-scores and Top-50 ranks within the augmented feature space are reported in Table 1.

The demographics-only RF showed substantially lower performance than the Fusion model, indicating that demographic variables alone could not reproduce the primary classification performance. Adding demographic variables to the Fusion feature set did not materially improve performance compared with Fusion alone. These results do not rule out demographic influence, especially given the age and BMI differences between ROA and non-ROA groups, but they suggest that the observed Fusion performance was not solely attributable to demographic covariates.

Figure S1. Model-level covariate sensitivity: Demographics-only RF, Fusion, and Fusion+Demographics performance on the locked test set. Metrics shown are F1, AUC, and specificity.

Table S1. Demographic variables' Fisher F-score and Top-50 rank within the augmented Fusion+Demographics feature space. Age and BMI were retained in the augmented feature space, whereas height and weight were not retained.

| Demographic | Fisher F-score | Top-50 rank |
| --- | --- | --- |
| Age | 13.42 | 1 |
| BMI | 5.26 | 12 |
| Weight | — | not retained |
| Height | — | not retained |
