# Supplementary material for: Radiographic Knee Osteoarthritis Identification Using Wearable Plantar Pressure: Secondary Cross-Sectional Analysis of Interpretable Framework Integrating Biomechanical Descriptors and the Multiscale Synergy Index
Source: JMIR Mhealth Uhealth. 2026 Sep 11;14:e101026. doi: 10.2196/101026 (PMC13566073; doi:10.2196/101026)
Supplement: Supplementary file 7 [file mhealth-v14-e101026-s007.docx]

This appendix provides the 2000-iteration bootstrap 95% confidence intervals for all locked test-set metrics and the paired DeLong comparisons. Both analyses were performed on the locked independent test set (n = 20).

Table S1. Locked test-set performance with 2000-iteration bootstrap 95% confidence intervals. Intervals were computed by resampling the 20 independent test subjects with replacement.

| Branch | Accuracy | F1 | AUC | Precision | Recall | Specificity |
| --- | --- | --- | --- | --- | --- | --- |
| PSI-only | 0.550 [0.350, 0.750] | 0.609 [0.333, 0.815] | 0.525 [0.263, 0.802] | 0.500 [0.250, 0.769] | 0.778 [0.500, 1.000] | 0.364 [0.091, 0.667] |
| Bio-only | 0.800 [0.600, 0.950] | 0.778 [0.500, 0.948] | 0.818 [0.583, 1.000] | 0.778 [0.462, 1.000] | 0.778 [0.455, 1.000] | 0.818 [0.571, 1.000] |
| Fusion | 0.850 [0.700, 1.000] | 0.842 [0.615, 1.000] | 0.818 [0.576, 1.000] | 0.800 [0.538, 1.000] | 0.889 [0.636, 1.000] | 0.818 [0.571, 1.000] |

Table S2. Paired DeLong comparisons on the locked independent test set (n = 20).

| Comparison | DeLong test (AUC) |
| --- | --- |
| Fusion vs Bio-only | AUC 0.818 vs 0.818; P>.99 |
| Fusion vs PSI-only | AUC 0.818 vs 0.525; P=.02 |
| Bio-only vs PSI-only | AUC 0.818 vs 0.525; P=.04 |
