# Supplementary material for: Radiographic Knee Osteoarthritis Identification Using Wearable Plantar Pressure: Secondary Cross-Sectional Analysis of Interpretable Framework Integrating Biomechanical Descriptors and the Multiscale Synergy Index
Source: JMIR Mhealth Uhealth. 2026 Sep 11;14:e101026. doi: 10.2196/101026 (PMC13566073; doi:10.2196/101026)
Supplement: Supplementary file 8 [file mhealth-v14-e101026-s008.docx]

This appendix provides the positive predictive value (PPV) and negative predictive value (NPV) of the Fusion, Bio-only, and PSI-only models across community ROA prevalence levels (5–30%). PPV and NPV were calculated from the sensitivity and specificity observed on the locked independent test set (n=20) using Bayes' theorem. Fusion maintained NPV ≥ 0.945 across the full prevalence range, providing supplementary clinical context for potential rule-out use. PPV scaled with prevalence, indicating that positive screening results should trigger confirmatory radiographic evaluation. This analysis is supplementary and should be interpreted with caution given the limited test-set size.

Table S1. Prevalence-adjusted positive predictive value (PPV) and negative predictive value (NPV) for Fusion, Bio-only, and PSI-only models across assumed community ROA prevalence levels. Values were derived from the sensitivity and specificity observed on the locked independent test set (n = 20) using Bayes’ theorem. This supplementary analysis should be interpreted cautiously given the limited test-set size.

| **Prevalence** | **Fusion PPV** | **Fusion NPV** | **Bio PPV** | **Bio NPV** | **PSI PPV** | **PSI NPV** |  |
| --- | --- | --- | --- | --- | --- | --- | --- |
| 5% | 0.205 | 0.993 | 0.184 | 0.986 | 0.061 | 0.969 |  |
| 10% | 0.352 | 0.985 | 0.322 | 0.971 | 0.120 | 0.937 |  |
| 20% | 0.550 | 0.967 | 0.517 | 0.937 | 0.234 | 0.868 |  |
| 30% | 0.677 | 0.945 | 0.647 | 0.896 | 0.344 | 0.793 |  |

**Figure S1. PPV (rule-in) and NPV (rule-out) as a function of community ROA prevalence (5–30%) for Fusion, Bio-only, and PSI-only models. NPV ≥ 0.945 for Fusion across the full range.**
